# Antibody-mediated prevention and treatment of HIV-1 infection

**DOI:** 10.1186/s12977-018-0455-9

**Published:** 2018-11-16

**Authors:** Henning Gruell, Florian Klein

**Affiliations:** 1 Laboratory of Experimental Immunology, Institute of Virology, University Hospital Cologne, Fürst-Pückler-Str. 56, 50935 Cologne, Germany; 2 German Center for Infection Research, Partner-Site Bonn-Cologne, Cologne, Germany; 3 Center for Molecular Medicine Cologne (CMMC), University of Cologne, Cologne, Germany

**Keywords:** HIV-1 broadly neutralizing antibodies, Immunotherapy, Passive immunization, (S)HIV animal models, Clinical trials, Neutralization, Fc-mediated activity

## Abstract

Novel broadly neutralizing antibodies targeting HIV-1 hold promise for their use in the prevention and treatment of HIV-1 infection. Pre-clinical results have encouraged the evaluation of these antibodies in healthy and HIV-1-infected humans. In first clinical trials, highly potent broadly neutralizing antibodies have demonstrated their safety and significant antiviral activity by reducing viremia and delaying the time to viral rebound in individuals interrupting antiretroviral therapy. While emerging antibody-resistant viral variants have indicated limitations of antibody monotherapy, strategies to enhance the efficacy of broadly neutralizing antibodies in humans are under investigation. These include the use of antibody combinations to prevent viral escape, antibody modifications to increase the half-life and the co-administration of latency-reversing agents to target the cellular reservoir of HIV-1. We provide an overview of the results of pre-clinical and clinical studies of broadly HIV-1 neutralizing antibodies, discuss their implications and highlight approaches for the ongoing advancement into humans.

## Background

Pathogen-specific antibodies are a hallmark of an effective immune response following infection or vaccination [1, 2]. Their development is the result of a cascade of events ranging from antigen uptake and presentation to B cell induction and antibody production [3]. Passive immunization, i.e., the administration of immunoglobulins, bypasses these steps. As such, it is an effective concept for immediate but transient protection from infections including hepatitis A, hepatitis B and rabies [4]. Moreover, the principle of antibody-mediated immunotherapy of infectious diseases has long been established by the use of toxin-specific antibodies to treat diphtheria or tetanus [5].

Advances in antibody production technology have enabled the development of highly active and specific clinical products. Antibodies have gained widespread medical use at an accelerating pace, with more than half of the > 70 available monoclonal antibodies and derived constructs having been approved over the span of the past 5 years [6]. Most of these antibodies are used in the treatment of malignant or autoimmune diseases. In contrast, approval of monoclonal antibodies that target infectious pathogens or pathogen-derived substances has been limited to antibodies against the respiratory syncytial virus and toxins produced by *Clostridium difficile* or *Bacillus anthracis*. Recently, the antibody ibalizumab has been approved for the treatment of multidrug-resistant HIV-1 infection [7]. While ibalizumab does not directly interact with the circulating virus or HIV-1-infected cells, it targets an extracellular CD4 domain and therefore interferes with the binding of HIV-1 to its primary receptor on target cells [7].

Despite being proposed early on [8], the idea of neutralizing antibody-mediated immunotherapy of HIV-1 infection was long abandoned because of limited activity in animal models and early clinical trials [9–14]. However, the isolation of highly potent broadly neutralizing anti-HIV-1 antibodies (bNAbs) has renewed enthusiasm about the potential application of these antibodies and resulted in numerous clinical trials investigating different concepts of bNAbs for HIV-1 infection.

## Main text

### First monoclonal HIV-1 neutralizing antibodies

Most HIV-1-infected individuals develop limited neutralizing serum activity. Accordingly, facing the enormous diversity of HIV-1, passive transfer of plasma or purified immunoglobulins from HIV-1-infected donors resulted in inconsistent or no detectable treatment effects in humans [15–18]. Similarly, the first monoclonal anti-HIV-1 antibodies failed to demonstrate significant antiviral effects in early clinical trials [19–23]. Limitations in potency and breadth remained for the first generation of broadly neutralizing antibodies [24–26]. However, proof-of-concept studies in non-human primates (NHPs) and humanized mice demonstrated that monoclonal antibodies can protect from infection with chimeric simian/human immunodeficiency virus (SHIV) and HIV-1 [27–41]. Nevertheless, these antibodies were not generally considered applicable for clinical use in HIV-1 prevention mainly because of an overall low neutralizing activity against the majority of viral strains. The bar for treatment of established infection proved even higher, as combinations of these early antibodies failed to significantly suppress viremia or prevent the development of resistance in animals and humans [9–14]. Thus, the results of these experiments reinforced the need for more potent antibodies that cover a wide spectrum of viral strains to facilitate bNAb-mediated prevention and treatment of HIV-1 infection.

### A new generation of antibodies targeting HIV-1

**Fig. 1:**
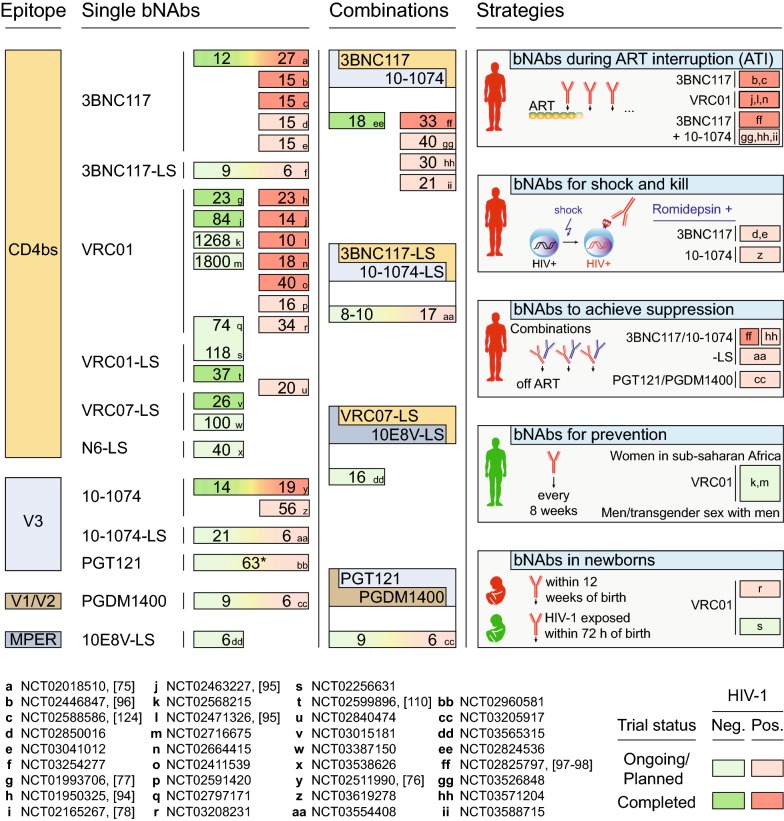
Clinical trials of new-generation broadly neutralizing antibodies. Numbers show (prospective) trial participants receiving bNAb(s). Letters encode the ClinicalTrials.gov study identifier. Healthy and HIV-1-infected individuals are indicated by green and red colors, respectively. Studies shown in dark colors have been completed, while studies shown in light colors are ongoing or not yet recruiting. Asterisk indicates that the number of participants includes those receiving placebo

Advances in antibody isolation and cloning methods, combined with the identification of subjects with exceptional neutralizing serum activity, resulted in the isolation of a new generation of anti-HIV-1 bNAbs [42–47]. These antibodies are orders of magnitude more potent than those isolated before and neutralize the majority of viral strains [48]. All bNAbs recognize the HIV-1 envelope glycoprotein (Env) by targeting defined vulnerable epitopes on its surface [49, 50]. Among them, antibodies against the CD4 binding site (3BNC117, VRC01) and the V3 loop (10-1074) have progressed beyond first-in-human trials to studies focusing on potential strategies for treatment and prevention of HIV-1 infection (Fig. 1). Additional antibodies targeting the CD4 binding site (N6-LS and VRC07-LS), the V3 loop (PGT121) or other epitopes (V1/V2 loop, PGDM1400; membrane proximal external region (MPER) of gp41, 10E8V-LS) are being investigated in early phase studies (Fig. 1). Indeed, more than 30 clinical trials have been initiated and will result in the enrollment of over 4000 study participants receiving one or a combination of novel broadly neutralizing antibodies (Fig. 1).

### Paving the way for prevention

Members of the new generation of highly potent bNAbs can protect from infection in parenteral, vaginal, rectal and/or oral viral challenge models [51–71]. In fact, bNAbs have been shown to prevent (S)HIV infection by high titer virus mucosal challenge across a number of animal studies investigating different bNAbs, viral strains and/or routes of transmission [55–67]. While mucosal application of high titer virus ensures robust infection after a single challenge, this model does not reflect the limited frequency of transmission seen for a single sexual contact or breastfeeding [72, 73]. Thus, it may underestimate the efficacy of bNAbs to prevent HIV-1 transmission in humans. Low-dose repeated mucosal challenge mimics clinical scenarios more closely. In such models, the administration of a single bNAb can significantly delay the time to infection [68–71]. For example, macaques intrarectally challenged with SHIV_AD8_ were protected from infection after a single administration of 10-1074, 3BNC117 or VRC01 until the median serum antibody concentrations declined to 0.17–1.83 µg/ml [70]. These levels were approximately 3-fold higher than the IC_50_s determined against the challenge virus in vitro [70]. Higher ratios of protective serum antibody concentrations and in vitro IC_50_s were observed for first-generation bNAbs in low-dose challenge models [68, 69]. However, these differences might be accounted for by the use of different virus strains, challenge routes and other conditions including the experimental thinning of epithelia in vaginal transmission models. Nevertheless, if the results from low-dose rectal challenge in NHPs hold true in humans, bNAb serum levels of 10 µg/ml are likely to be sufficient to prevent infection from a large fraction of circulating viruses [74]. When infused intravenously (i.v.), 3BNC117, VRC01 and 10-1074 showed mean half-lives of 11–24 days in healthy individuals [75–79]. Following an infusion of either antibody at a dose of 20–30 mg/kg, bNAb levels of > 10 µg/ml were measured for approximately 8–16 weeks [75–77]. Importantly, this period can be substantially extended by antibody modifications discussed below.

In contrast to the challenge with selected monoclonal viruses in animal models, humans are exposed to a wide range of viral strains with different antibody sensitivities. Thus, whether bNAbs can afford a meaningful degree of protection from HIV-1 infection in humans can only be demonstrated in clinical trials. Two large placebo-controlled studies aim to answer this question using the CD4 binding site antibody VRC01. To this end, VRC01 is given at 10 or 30 mg/kg every 2 months to individuals at high risk of acquiring HIV-1 infection (NCT02568215, women living in sub-Saharan Africa; NCT02716675, men and transgender persons who have sex with men) [78, 80]. These are critical proof-of-concept studies, however, more potent antibodies or bNAb combinations may provide more effective options for prevention.

Passively administered bNAbs need to be applied repeatedly to maintain levels above a threshold concentration required for effective protection. Transgenic bNAb expression could be a feasible approach to overcome this limitation. For example, administration of adeno-associated viruses (AAVs) can result in sustained transgene expression, and their safety has been demonstrated throughout a number of clinical trials [81]. In humanized mice, AAV-mediated bNAb expression can protect from HIV-1 infection by repeated mucosal viral challenge [82, 83]. To investigate this concept of vectored immunoprophylaxis in humans, phase I studies of AAVs encoding for the anti-V1/V2 loop antibody PG9 or the CD4 binding site antibody VRC07 have been initiated (NCT01937455, NCT03374202).

### Gaining traction for treatment

The identification of novel highly potent bNAbs prompted the re-assessment of antibody-mediated therapy of established infection in humanized mice and non-human primates [67, 84–91]. Treatment of HIV-1-infected mice with single bNAbs resulted in the rapid emergence of mutations at antibody target sites that were associated with viral rebound [84–86, 88, 89]. However, in contrast to earlier bNAbs, combinations of new-generation bNAbs targeting non-overlapping epitopes effectively maintained suppression of viremia [84, 85, 87]. Sequence analyses of viruses obtained during and after treatment demonstrated the lack of concurrent escape mutations at all antibody target residues [84, 87]. Thus, similar to combinations of classical antiretroviral drugs, combination antibody therapy can prevent the development of viral resistance in humanized mice.

In SHIV-infected non-human primates, the duration and magnitude of viral suppression during bNAb monotherapy appeared to be more pronounced than in humanized mice [67, 90, 91]. These differences might be explained by the fully functional immune system that is present in non-human primates but absent in humanized mice. Indeed, host immunity does play a critical role for the antiviral activity of HIV-1 neutralizing antibodies as demonstrated for Fc-mediated effector functions in both animal models [51, 52, 92, 93]. Underlining the impact on bNAb-mediated antiviral activity, the combination of bNAbs in NHPs prolonged suppression of sensitive SHIV strains and limited the development of viral resistance compared to single bNAbs [67].

### bNAb monotherapy in humans

Early phase clinical trials started translating these findings to HIV-1-infected humans, beginning with the CD4 binding site antibodies 3BNC117 [75] and VRC01 [94], and followed by the V3 loop antibody 10-1074 [76]. Importantly, the administration of these antibodies was found to be safe and very well tolerated across all trials completed to date [75–78, 94–98]. Moreover, infusion of either 3BNC117, VRC01 or 10-1074 at a dose of 30–40 mg/kg to sensitive viremic individuals resulted in rapid reduction of viremia by an average of 1.5, 1.1 and 1.5 log_10_, respectively [75, 76, 94]. However, suppression of viral load below the limit of detection was only rarely achieved, and viral rebound generally occurred within 4 weeks. Rebound was associated with increased resistance against the administered bNAbs in most cases, although the extent differed between antibodies. Following the administration of the V3 loop antibody 10-1074, a rapid selection of fully resistant escape variants was observed in all study participants [76]. In contrast, infusion of the CD4 binding site antibodies 3BNC117 or VRC01 resulted in a general trend of reduced viral sensitivity, but was not consistently associated with the development of full resistance [75, 94]. For example, in six sensitive viremic individuals receiving 3BNC117 at a single dose of 10 or 30 mg/kg, autologous culture outgrowth viruses remained partially sensitive to 3BNC117 with an increase of the geometric mean IC_50_ against 3BNC117 from 0.2 µg/ml to only 1.7 µg/ml [75]. These findings might indicate that antibodies with similar effects on the viral load differ in their capacity to restrict viral escape. Importantly, the envelope protein targeted by broadly neutralizing antibodies has a critical function in the viral replication cycle, and escape from some bNAbs has been associated with reductions in viral fitness [76, 99, 100]. For example, in vitro studies of naturally occurring mutations that confer resistance against the CD4 binding site antibody VRC01 showed a negative impact on the viral replicative capacity that could, however, be restored through compensatory mutations [99].

Compared to active viral replication in viremic individuals, ART-mediated suppression at the onset of bNAb therapy may impede the development of escape mutations. In agreement with this idea, single antibodies were more effective in maintaining viral suppression in HIV-1-infected humanized mice following an initial period of antiretroviral therapy [85]. To test this concept in humans, monotherapy with the bNAb 3BNC117 or VRC01 was administered to HIV-1-infected individuals undergoing analytical treatment interruption (ATI) of antiretroviral therapy [95, 96]. While 3BNC117 or VRC01 delayed the time to viral rebound to 10 or 4 weeks, respectively, rebound did occur in the presence of high bNAb serum levels in most cases and was associated with increased antibody resistance [95, 96].

Taken together, first clinical trials demonstrated the safety and significant antiviral activity of novel broadly neutralizing antibodies targeting HIV-1. However, the emergence of viral escape variants has highlighted the limitations of antibody monotherapy.

### Combining antibodies for HIV-1 therapy

Based on the well-established concept of preventing viral escape through combinations of antiretroviral drugs and similar results for bNAbs in pre-clinical studies, clinical trials that combine new-generation bNAbs were initiated (Fig. 1). In the first study, the combination of 3BNC117 and 10-1074 showed similar safety and pharmacokinetic profiles to either antibody alone [97, 98]. In four viremic individuals determined to be infected with viruses sensitive to both antibodies, treatment with up to three infusions of 3BNC117 and 10-1074 resulted in an average drop in viremia of 2.0 log_10_ copies/ml [97]. In most of these individuals, reduced viral loads were maintained for as long as both of the administered antibodies were detectable in the serum (8–12 weeks after the last antibody infusion) [97]. Moreover, in contrast to 10-1074 monotherapy [76], antibody escape did not develop in all instances [97]. However, despite the significant reduction of the viral load, full suppression was only achieved in study participants with relatively low levels of viremia (below 3000 copies/ml) [97].

More pronounced effects were observed in individuals infected with antibody-sensitive viruses undergoing ATI. These participants received the antibody combination at 0, 3 and 6 weeks after stopping ART. In contrast to the time to rebound without intervention (2.4 weeks, historical controls) or 3BNC117 monotherapy (9.9 weeks) [96], the combination of 3BNC117 and 10-1074 maintained viral suppression for a median of 21 weeks or nearly 4 months after the last antibody infusion [98].

Of note, 12 out of 13 individuals (4 viremic, 9 undergoing ART interruption) with viruses sensitive to 3BNC117 and 10-1074 did not experience viral rebound as long as both antibodies had serum concentrations above 10 µg/ml [97, 98]. Thus, combinations of new generation bNAbs at sufficient antibody concentrations are effective in maintaining viral suppression in humans infected with sensitive viruses.

### Preparing for practice

Antiretroviral drugs are highly effective in treating HIV-1 infection and reducing the risk of infection when used as pre-exposure prophylaxis. Moreover, they are well-established, easily distributable, increasingly available in generic form and long-acting injectable drugs are at the final stages of development [101]. Clinical implementation of broadly neutralizing antibodies will therefore not only require safe and highly active products, but also depend on the ease of administration, cost-effectiveness and well-designed strategies for their use.

Neutralizing potency and breadth are the most obvious prerequisites for the activity of bNAbs in vivo. In addition, the capacity to restrict viral escape is likely to be an equally critical parameter for the efficacy of bNAbs. Results from bNAb monotherapy trials indicate that combinations of antibodies are required to reduce the development of viral resistance. All current combination studies target two non-overlapping epitopes (CD4 binding site and V3 loop; V1/V2 loop and V3 loop; CD4 binding site and MPER of gp41) (Fig. 1). Strategies that target more than two epitopes may further impede the development of viral resistance as well as increase the probability of capturing partially resistant variants. As an alternative to antibody combinations, bi- or tri-specific antibody-like molecules have been demonstrated to have similar or enhanced antiviral activity and clinical trials are about to be initiated [53, 64, 102, 103]. Finally, combinations of antibodies that bind to overlapping epitopes may restrict escape pathways for the given target [87]. This may be particularly effective for antibody target sites that are limited in their capacity to accommodate mutations.

Viral strains differ in their sensitivity to antibodies. Moreover, the HIV-1 envelope protein diversifies in response to the autologous immune response and different viral variants co-exist within one person. Thus, the selection of bNAbs needs to be tailored to an individual’s viral quasispecies to prevent treatment failure. Phenotypic sensitivity assays of viruses derived from bulk T cell outgrowth cultures fail to detect pre-existing resistant variants in a relevant number of cases [75, 76, 96–98]. Limiting dilution outgrowth assays increase the sensitivity, however, they are time-consuming and costly [98, 104]. In contrast to phenotypic testing, antiretroviral therapy is mostly guided by prediction models based on viral sequences [105]. Similar approaches based on *env* sequences are under development but will need to be confirmed in prospective settings [106, 107].

While terminal elimination half-lives of most antiretroviral drugs range between a few hours to 2 days, the half-lives of bNAbs are measured in weeks and result in long periods of effective plasma concentrations after a single administration. Notably, these periods can be further extended by modifications of the antibody Fc domains that enhance the affinity to the neonatal Fc receptor [108]. For example, the M428L and N434S (“LS”) mutations prolong antibody half-life without compromising antigen-binding or other Fc-mediated functions [109]. Indeed, the LS variant of VRC01 demonstrated a half-life of ≈ 70 days in healthy individuals, which is a nearly 5-fold increase compared to the unmodified VRC01 [110]. The extended half-life of LS variants is also reflected in prolonged protective activity in pre-clinical studies [70, 71]. Thus, LS-modified bNAbs may facilitate dosing every few weeks to several months for treatment or even less frequently for prevention.

Compared to the ease of oral application of most regular antiretroviral drugs, the intravenous route employed in most clinical trials of bNAbs can be impractical. Subcutaneous (s.c.) injection, however, allows for easy (self-)administration and bNAbs have shown similar half-lives when given s.c. or i.v. [77, 78, 94, 110]. While antibody peak concentrations are lower after s.c. application and injection volumes pose restrictions, these limitations can be compensated by advances in antibody formulations and extended half-lives. Finally, antibodies can be administered topically and vaginal application of anti-HIV-1 bNAbs was generally safe in clinical trials [111, 112]. In proof-of-concept studies, this strategy protected animals from infection [113–115]. While these findings would need to be confirmed in humans, adherence to repeated and timely administration is a critical and potentially limiting factor for the efficacy of topically applied antibodies [116].

### Going forward and beyond neutralization

Despite substantial differences in their modes of action, both antiretroviral drugs and bNAbs suppress viremia. Thus, bNAbs may provide a treatment option for individuals infected with ART-resistant viruses as well as for individuals suffering from side effects or toxicities caused by ART. Effective ART with three active drugs leads to rapid reduction of high viral loads to levels undetectable by standard clinical assays. Whether this can be equally achieved by bNAb combinations remains to be determined. However, first results suggest that bNAb-mediated therapy is particularly effective in individuals with low or suppressed starting viral loads [95, 96, 98]. Therefore, an initial phase of ART followed by bNAb-mediated therapy is a promising strategy for long-term control of the virus. For all of these approaches, as well as for the potential application of bNAbs for pre-exposure prophylaxis, the long half-life of bNAbs can significantly reduce the burden of daily medication and the need for meticulous adherence.

Broadly neutralizing antibodies differ from classical antiretroviral drugs in that they directly target the circulating virus, recognize HIV-1-infected cells expressing HIV-1 Env and can engage with the host immune system. Indeed, Fc-mediated interactions have been demonstrated to be important for effective bNAb-mediated (S)HIV control and prevention in animal models [51, 52, 92, 93]. In addition, passively administered bNAbs can influence the extent of the autologous antiviral immune response. For example, a single infusion of 3BNC117 was associated with the development of enhanced host neutralizing antibody activity in HIV-1-infected individuals [117], corroborating similar observations made in SHIV-infected animals [118–121]. Moreover, bNAb therapy has been associated with enhancement of cellular immune responses [93, 122, 123]. Notably, administration of bNAbs 3BNC117 and 10-1074 during early SHIV-infection resulted in long-term viral suppression. As demonstrated by rapid viral rebound after CD8^+^ T cell depletion, viral suppression was effectively mediated by T cells when the antibodies were no longer detectable in the serum [123]. Whether these effects can be exploited for an improvement of clinical outcomes in humans remains to be determined. In particular, the potential effects of bNAbs given during acute or early infection will be important to investigate in clinical trials.

Additionally, bNAbs contribute to the elimination of HIV-1-infected cells [93]. This activity may also extend to the clearance of viral foci established early after exposure [58, 66]. The capacity of antibodies to mediate the elimination of HIV-1-infected cells will become particularly relevant in strategies that target the HIV-1 reservoir. However, no significant changes in the size of the circulating latent reservoir were observed after the infusion of 3BNC117 or VRC01 to individuals on ongoing suppressive ART, or after the combined administration of 3BNC117 and 10-1074 during interruption of ART [94, 98, 124]. However, these studies had relatively short observation periods (up to a few months), involved only a low number of antibody infusions and mainly included individuals with chronic HIV-1 infection. All of these factors may have limited bNAb-mediated effects on the viral reservoir or their detection.

Stimulation and induction of HIV-1 Env expression on the surface of latently infected cells make them an approachable target for bNAbs that can mediate their clearance by engaging the host immune system (so-called shock and kill approach). Indeed, when bNAbs were combined with latency-reversing agents (LRAs), long-term viral suppression was observed in a fraction of (S)HIV-infected humanized mice and macaques [52, 125]. To investigate this concept in humans, the histone deacetylase inhibitor romidepsin is being studied in combination with 3BNC117 (NCT02850016, NCT03041012) as well as in combination with 10-1074 and experimental therapeutic vaccines (NCT03619278). When given to ART-treated individuals, romidepsin has been shown to result in transient viremia [126]. While the effects of romidepsin given in combination with bNAbs will be important to determine, latency-reversing strategies will likely require further optimization such as combinations of LRAs or use of additional drugs (e.g., interferon alpha [127]).

## Conclusions

Newly identified highly potent broadly neutralizing anti-HIV-1 antibodies have rapidly advanced from pre-clinical experiments to clinical trials that have demonstrated their safety and significant antiviral activity. Moreover, these studies have improved our understanding on how to establish bNAb interventions for clinical practice.

Preventing the development of viral resistance is a key factor for effective bNAb-mediated therapy and, similar to antiretroviral drugs, combinations of antibodies or poly-specific antibody variants will be required to increase the barrier for HIV-1 escape. In determining optimal combination partners, factors beyond mere HIV-1 coverage will be relevant and are likely to include the efficacy in restricting viral escape pathways. Equally important, improved and reliable screening methods are needed to guide clinicians in bNAb selection and the identification of candidates for effective bNAb therapy.

Ongoing and planned trials will aid in the development of effective treatment and prevention strategies. In particular, bNAbs appear to be especially useful in maintaining viral suppression in a setting of ART interruption. Moreover, antibodies may contribute to a reduction in the reservoir of HIV-1-infected cells as part of future cure strategies. Finally, modified antibody variants with substantially increased half-lives facilitate infrequent dosing of antibodies, and improved formulations will allow for alternatives to i.v. application that will be of particular interest for the use of bNAbs in prevention.

By limiting disease progression and reducing viral transmission, antiretroviral drugs have profoundly affected the course of the HIV-1 pandemic. With highly potent broadly neutralizing antibodies now demonstrating their impressive potential in pre-clinical and clinical settings, novel agents for the treatment and prevention of HIV-1 infection have come into the reach of clinical reality. Delineating the critical factors for successful application of bNAbs will be essential to exploit the unique capabilities of antibodies to benefit HIV-1-infected patients and those at risk of acquiring HIV-1 infection.

## Abbreviations

AAV: adeno-associated virus
ART: antiretroviral therapy
ATI: analytical treatment interruption
bNAb: broadly neutralizing antibody
Env: envelope protein
Fc: fragment crystallizable
HIV: human immunodeficiency virus
IC: inhibitory concentration
iv: intravenously
LRA: latency-reversing agent
MPER: membrane proximal external region
NHP: non-human primate
sc: subcutaneously
SHIV: simian/human chimeric immunodeficiency virus

## References

[1] Plotkin SA (2010). Correlates of protection induced by vaccination. Clin Vaccine Immunol.

[2] Ahmed R, Gray D (1996). Immunological memory and protective immunity: understanding their relation. Science.

[3] Rajewsky K (1996). Clonal selection and learning in the antibody system. Nature.

[4] Keller MA, Stiehm ER (2000). Passive immunity in prevention and treatment of infectious diseases. Clin Microbiol Rev.

[5] von Behring E, Kitasato S (1890). Ueber das Zustandekommen der Diphtherie-Immunität und der Tetanus-Immunität bei Thieren. Dtsch Med Wochenschr.

[6] Kaplon H, Reichert JM (2018). Antibodies to watch in 2018. MAbs.

[7] Emu B, Fessel J, Schrader S, Kumar P, Richmond G, Win S, Weinheimer S, Marsolais C, Lewis S (2018). Phase 3 study of ibalizumab for multidrug-resistant HIV-1. N Engl J Med.

[8] Karpas A, Gillson W, Bevan PC, Oates JK (1985). Lytic infection by British AIDS virus and development of rapid cell test for antiviral antibodies. Lancet.

[9] Poignard P, Sabbe R, Picchio GR, Wang M, Gulizia RJ, Katinger H, Parren PW, Mosier DE, Burton DR (1999). Neutralizing antibodies have limited effects on the control of established HIV-1 infection in vivo. Immunity.

[10] Armbruster C, Stiegler GM, Vcelar BA, Jager W, Koller U, Jilch R, Ammann CG, Pruenster M, Stoiber H, Katinger HW (2004). Passive immunization with the anti-HIV-1 human monoclonal antibody (hMAb) 4E10 and the hMAb combination 4E10/2F5/2G12. J Antimicrob Chemother.

[11] Armbruster C, Stiegler GM, Vcelar BA, Jager W, Michael NL, Vetter N, Katinger HW (2002). A phase I trial with two human monoclonal antibodies (hMAb 2F5, 2G12) against HIV-1. AIDS.

[12] Stiegler G, Armbruster C, Vcelar B, Stoiber H, Kunert R, Michael NL, Jagodzinski LL, Ammann C, Jager W, Jacobson J (2002). Antiviral activity of the neutralizing antibodies 2F5 and 2G12 in asymptomatic HIV-1-infected humans: a phase I evaluation. AIDS.

[13] Trkola A, Kuster H, Rusert P, Joos B, Fischer M, Leemann C, Manrique A, Huber M, Rehr M, Oxenius A (2005). Delay of HIV-1 rebound after cessation of antiretroviral therapy through passive transfer of human neutralizing antibodies. Nat Med.

[14] Mehandru S, Vcelar B, Wrin T, Stiegler G, Joos B, Mohri H, Boden D, Galovich J, Tenner-Racz K, Racz P (2007). Adjunctive passive immunotherapy in human immunodeficiency virus type 1-infected individuals treated with antiviral therapy during acute and early infection. J Virol.

[15] Levy J, Youvan T, Lee ML (1994). Passive hyperimmune plasma therapy in the treatment of acquired immunodeficiency syndrome: results of a 12-month multicenter double-blind controlled trial. The Passive Hyperimmune Therapy Study Group. Blood.

[16] Vittecoq D, Chevret S, Morand-Joubert L, Heshmati F, Audat F, Bary M, Dusautoir T, Bismuth A, Viard JP, Barre-Sinoussi F (1995). Passive immunotherapy in AIDS: a double-blind randomized study based on transfusions of plasma rich in anti-human immunodeficiency virus 1 antibodies vs. transfusions of seronegative plasma. Proc Natl Acad Sci USA.

[17] Jacobson JM, Colman N, Ostrow NA, Simson RW, Tomesch D, Marlin L, Rao M, Mills JL, Clemens J, Prince AM (1993). Passive immunotherapy in the treatment of advanced human immunodeficiency virus infection. J Infect Dis.

[18] Stiehm ER, Lambert JS, Mofenson LM, Bethel J, Whitehouse J, Nugent R, Moye J, Glenn Fowler M, Mathieson BJ, Reichelderfer P (1999). Efficacy of zidovudine and human immunodeficiency virus (HIV) hyperimmune immunoglobulin for reducing perinatal HIV transmission from HIV-infected women with advanced disease: results of Pediatric AIDS Clinical Trials Group protocol 185. J Infect Dis.

[19] Cavacini LA, Samore MH, Gambertoglio J, Jackson B, Duval M, Wisnewski A, Hammer S, Koziel C, Trapnell C, Posner MR (1998). Phase I study of a human monoclonal antibody directed against the CD4-binding site of HIV type 1 glycoprotein 120. AIDS Res Hum Retroviruses.

[20] Dezube BJ, Doweiko JP, Proper JA, Conway B, Hwang L, Terada M, Leece BA, Ohno T, Mastico RA (2004). Monoclonal antibody hNM01 in HIV-infected patients: a phase I study. J Clin Virol.

[21] Hinkula J, Bratt G, Gilljam G, Nordlund S, Broliden PA, Holmberg V, Olausson-Hansson E, Albert J, Sandstrom E, Wahren B (1994). Immunological and virological interactions in patients receiving passive immunotherapy with HIV-1 neutralizing monoclonal antibodies. J Acquir Immune Defic Syndr.

[22] Matsushita S, Yoshimura K, Ramirez KP, Pisupati J, Murakami T, Group KDS (2015). Passive transfer of neutralizing mAb KD-247 reduces plasma viral load in patients chronically infected with HIV-1. AIDS.

[23] Gunthard HF, Gowland PL, Schupbach J, Fung MS, Boni J, Liou RS, Chang NT, Grob P, Graepel P, Braun DG (1994). A phase I/IIA clinical study with a chimeric mouse-human monoclonal antibody to the V3 loop of human immunodeficiency virus type 1 gp120. J Infect Dis.

[24] Posner MR, Hideshima T, Cannon T, Mukherjee M, Mayer KH, Byrn RA (1991). An IgG human monoclonal-antibody that reacts with HIV-1/Gp120, inhibits virus binding to cells, and neutralizes infection. J Immunol.

[25] Buchacher A, Predl R, Strutzenberger K, Steinfellner W, Trkola A, Purtscher M, Gruber G, Tauer C, Steindl F, Jungbauer A (1994). Generation of human monoclonal antibodies against HIV-1 proteins; electrofusion and Epstein-Barr virus transformation for peripheral blood lymphocyte immortalization. AIDS Res Hum Retroviruses.

[26] Burton DR, Barbas CF, Persson MA, Koenig S, Chanock RM, Lerner RA (1991). A large array of human monoclonal antibodies to type 1 human immunodeficiency virus from combinatorial libraries of asymptomatic seropositive individuals. Proc Natl Acad Sci USA.

[27] Safrit JT, Fung MSC, Andrews CA, Braun DG, Sun WNC, Chang TW, Koup RA (1993). Hu-Pbl-Scid mice can be protected from HIV-1 infection by passive transfer of monoclonal-antibody to the principal neutralizing determinant of envelope gp120. AIDS.

[28] Gauduin MC, Safrit JT, Weir R, Fung MS, Koup RA (1995). Pre- and postexposure protection against human immunodeficiency virus type 1 infection mediated by a monoclonal antibody. J Infect Dis.

[29] Emini EA, Schleif WA, Nunberg JH, Conley AJ, Eda Y, Tokiyoshi S, Putney SD, Matsushita S, Cobb KE, Jett CM (1992). Prevention of HIV-1 infection in chimpanzees by gp120 V3 domain-specific monoclonal antibody. Nature.

[30] Gauduin MC, Parren PW, Weir R, Barbas CF, Burton DR, Koup RA (1997). Passive immunization with a human monoclonal antibody protects hu-PBL-SCID mice against challenge by primary isolates of HIV-1. Nat Med.

[31] Parren PW, Ditzel HJ, Gulizia RJ, Binley JM, Barbas CF, Burton DR, Mosier DE (1995). Protection against HIV-1 infection in hu-PBL-SCID mice by passive immunization with a neutralizing human monoclonal antibody against the gp120 CD4-binding site. AIDS.

[32] Mascola JR, Lewis MG, Stiegler G, Harris D, VanCott TC, Hayes D, Louder MK, Brown CR, Sapan CV, Frankel SS (1999). Protection of Macaques against pathogenic simian/human immunodeficiency virus 89.6PD by passive transfer of neutralizing antibodies. J Virol.

[33] Baba TW, Liska V, Hofmann-Lehmann R, Vlasak J, Xu W, Ayehunie S, Cavacini LA, Posner MR, Katinger H, Stiegler G (2000). Human neutralizing monoclonal antibodies of the IgG1 subtype protect against mucosal simian-human immunodeficiency virus infection. Nat Med.

[34] Deruaz M, Moldt B, Le KM, Power KA, Vrbanac VD, Tanno S, Ghebremichael MS, Allen TM, Tager AM, Burton DR, Luster AD (2016). Protection of humanized mice from repeated intravaginal HIV challenge by passive immunization: a model for studying the efficacy of neutralizing antibodies in vivo. J Infect Dis.

[35] Burton DR, Hessell AJ, Keele BF, Klasse PJ, Ketas TA, Moldt B, Dunlop DC, Poignard P, Doyle LA, Cavacini L (2011). Limited or no protection by weakly or nonneutralizing antibodies against vaginal SHIV challenge of macaques compared with a strongly neutralizing antibody. Proc Natl Acad Sci USA.

[36] Hessell AJ, Rakasz EG, Poignard P, Hangartner L, Landucci G, Forthal DN, Koff WC, Watkins DI, Burton DR (2009). Broadly neutralizing human anti-HIV antibody 2G12 is effective in protection against mucosal SHIV challenge even at low serum neutralizing titers. PLoS Pathog.

[37] Hessell AJ, Rakasz EG, Tehrani DM, Huber M, Weisgrau KL, Landucci G, Forthal DN, Koff WC, Poignard P, Watkins DI, Burton DR (2010). Broadly neutralizing monoclonal antibodies 2F5 and 4E10 directed against the human immunodeficiency virus type 1 gp41 membrane-proximal external region protect against mucosal challenge by simian-human immunodeficiency virus SHIVBa-L. J Virol.

[38] Mascola JR, Stiegler G, VanCott TC, Katinger H, Carpenter CB, Hanson CE, Beary H, Hayes D, Frankel SS, Birx DL, Lewis MG (2000). Protection of macaques against vaginal transmission of a pathogenic HIV-1/SIV chimeric virus by passive infusion of neutralizing antibodies. Nat Med.

[39] Parren PW, Marx PA, Hessell AJ, Luckay A, Harouse J, Cheng-Mayer C, Moore JP, Burton DR (2001). Antibody protects macaques against vaginal challenge with a pathogenic R5 simian/human immunodeficiency virus at serum levels giving complete neutralization in vitro. J Virol.

[40] Ferrantelli F, Hofmann-Lehmann R, Rasmussen RA, Wang T, Xu W, Li PL, Montefiori DC, Cavacini LA, Katinger H, Stiegler G (2003). Post-exposure prophylaxis with human monoclonal antibodies prevented SHIV89.6P infection or disease in neonatal macaques. AIDS.

[41] Hofmann-Lehmann R, Vlasak J, Rasmussen RA, Smith BA, Baba TW, Liska V, Ferrantelli F, Montefiori DC, McClure HM, Anderson DC (2001). Postnatal passive immunization of neonatal macaques with a triple combination of human monoclonal antibodies against oral simian-human immunodeficiency virus challenge. J Virol.

[42] Walker LM, Phogat SK, Chan-Hui PY, Wagner D, Phung P, Goss JL, Wrin T, Simek MD, Fling S, Mitcham JL (2009). Broad and potent neutralizing antibodies from an African donor reveal a new HIV-1 vaccine target. Science.

[43] Simek MD, Rida W, Priddy FH, Pung P, Carrow E, Laufer DS, Lehrman JK, Boaz M, Tarragona-Fiol T, Miiro G (2009). Human immunodeficiency virus type 1 elite neutralizers: individuals with broad and potent neutralizing activity identified by using a high-throughput neutralization assay together with an analytical selection algorithm. J Virol.

[44] Scheid JF, Mouquet H, Feldhahn N, Walker BD, Pereyra F, Cutrell E, Seaman MS, Mascola JR, Wyatt RT, Wardemann H, Nussenzweig MC (2009). A method for identification of HIV gp140 binding memory B cells in human blood. J Immunol Methods.

[45] Scheid JF, Mouquet H, Ueberheide B, Diskin R, Klein F, Oliveira TY, Pietzsch J, Fenyo D, Abadir A, Velinzon K (2011). Sequence and structural convergence of broad and potent HIV antibodies that mimic CD4 binding. Science.

[46] Walker LM, Huber M, Doores KJ, Falkowska E, Pejchal R, Julien JP, Wang SK, Ramos A, Chan-Hui PY, Moyle M (2011). Broad neutralization coverage of HIV by multiple highly potent antibodies. Nature.

[47] Wu X, Yang ZY, Li Y, Hogerkorp CM, Schief WR, Seaman MS, Zhou T, Schmidt SD, Wu L, Xu L (2010). Rational design of envelope identifies broadly neutralizing human monoclonal antibodies to HIV-1. Science.

[48] Walker LM, Burton DR (2018). Passive immunotherapy of viral infections: ‘super-antibodies’ enter the fray. Nat Rev Immunol.

[49] Burton DR, Hangartner L (2016). Broadly neutralizing antibodies to HIV and their role in vaccine design. Annu Rev Immunol.

[50] West AP, Scharf L, Scheid JF, Klein F, Bjorkman PJ, Nussenzweig MC (2014). Structural insights on the role of antibodies in HIV-1 vaccine and therapy. Cell.

[51] Bournazos S, Klein F, Pietzsch J, Seaman MS, Nussenzweig MC, Ravetch JV (2014). Broadly neutralizing anti-HIV-1 antibodies require Fc effector functions for in vivo activity. Cell.

[52] Halper-Stromberg A, Lu CL, Klein F, Horwitz JA, Bournazos S, Nogueira L, Eisenreich TR, Liu C, Gazumyan A, Schaefer U (2014). Broadly neutralizing antibodies and viral inducers decrease rebound from HIV-1 latent reservoirs in humanized mice. Cell.

[53] Huang Y, Yu J, Lanzi A, Yao X, Andrews CD, Tsai L, Gajjar MR, Sun M, Seaman MS, Padte NN, Ho DD (2016). Engineered bispecific antibodies with exquisite HIV-1-neutralizing activity. Cell.

[54] Pietzsch J, Gruell H, Bournazos S, Donovan BM, Klein F, Diskin R, Seaman MS, Bjorkman PJ, Ravetch JV, Ploss A, Nussenzweig MC (2012). A mouse model for HIV-1 entry. Proc Natl Acad Sci USA.

[55] Julg B, Liu PT, Wagh K, Fischer WM, Abbink P, Mercado NB, Whitney JB, Nkolola JP, McMahan K, Tartaglia LJ (2017). Protection against a mixed SHIV challenge by a broadly neutralizing antibody cocktail. Sci Transl Med.

[56] Julg B, Sok D, Schmidt SD, Abbink P, Newman RM, Broge T, Linde C, Nkolola J, Le K, Su D (2017). Protective efficacy of broadly neutralizing antibodies with incomplete neutralization activity against simian-human immunodeficiency virus in rhesus monkeys. J Virol.

[57] Julg B, Tartaglia LJ, Keele BF, Wagh K, Pegu A, Sok D, Abbink P, Schmidt SD, Wang K, Chen X (2017). Broadly neutralizing antibodies targeting the HIV-1 envelope V2 apex confer protection against a clade C SHIV challenge. Sci Transl Med.

[58] Liu J, Ghneim K, Sok D, Bosche WJ, Li Y, Chipriano E, Berkemeier B, Oswald K, Borducchi E, Cabral C (2016). Antibody-mediated protection against SHIV challenge includes systemic clearance of distal virus. Science.

[59] Moldt B, Le KM, Carnathan DG, Whitney JB, Schultz N, Lewis MG, Borducchi EN, Smith KM, Mackel JJ, Sweat SL (2016). Neutralizing antibody affords comparable protection against vaginal and rectal simian/human immunodeficiency virus challenge in macaques. AIDS.

[60] Moldt B, Rakasz EG, Schultz N, Chan-Hui PY, Swiderek K, Weisgrau KL, Piaskowski SM, Bergman Z, Watkins DI, Poignard P, Burton DR (2012). Highly potent HIV-specific antibody neutralization in vitro translates into effective protection against mucosal SHIV challenge in vivo. Proc Natl Acad Sci USA.

[61] Pegu A, Yang ZY, Boyington JC, Wu L, Ko SY, Schmidt SD, McKee K, Kong WP, Shi W, Chen X (2014). Neutralizing antibodies to HIV-1 envelope protect more effectively in vivo than those to the CD4 receptor. Sci Transl Med.

[62] Rudicell RS, Kwon YD, Ko SY, Pegu A, Louder MK, Georgiev IS, Wu X, Zhu J, Boyington JC, Chen X (2014). Enhanced potency of a broadly neutralizing HIV-1 antibody in vitro improves protection against lentiviral infection in vivo. J Virol.

[63] Shingai M, Donau OK, Plishka RJ, Buckler-White A, Mascola JR, Nabel GJ, Nason MC, Montefiori D, Moldt B, Poignard P (2014). Passive transfer of modest titers of potent and broadly neutralizing anti-HIV monoclonal antibodies block SHIV infection in macaques. J Exp Med.

[64] Xu L, Pegu A, Rao E, Doria-Rose N, Beninga J, McKee K, Lord DM, Wei RR, Deng G, Louder M (2017). Trispecific broadly neutralizing HIV antibodies mediate potent SHIV protection in macaques. Science.

[65] Saunders KO, Pegu A, Georgiev IS, Zeng M, Joyce MG, Yang ZY, Ko SY, Chen X, Schmidt SD, Haase AT (2015). Sustained delivery of a broadly neutralizing antibody in nonhuman primates confers long-term protection against simian/human immunodeficiency virus infection. J Virol.

[66] Hessell AJ, Jaworski JP, Epson E, Matsuda K, Pandey S, Kahl C, Reed J, Sutton WF, Hammond KB, Cheever TA (2016). Early short-term treatment with neutralizing human monoclonal antibodies halts SHIV infection in infant macaques. Nat Med.

[67] Shingai M, Nishimura Y, Klein F, Mouquet H, Donau OK, Plishka R, Buckler-White A, Seaman M, Piatak M, Lifson JD (2013). Antibody-mediated immunotherapy of macaques chronically infected with SHIV suppresses viraemia. Nature.

[68] Moldt B, Shibata-Koyama M, Rakasz EG, Schultz N, Kanda Y, Dunlop DC, Finstad SL, Jin C, Landucci G, Alpert MD (2012). A nonfucosylated variant of the anti-HIV-1 monoclonal antibody b12 has enhanced FcgammaRIIIa-mediated antiviral activity in vitro but does not improve protection against mucosal SHIV challenge in macaques. J Virol.

[69] Hessell AJ, Poignard P, Hunter M, Hangartner L, Tehrani DM, Bleeker WK, Parren PW, Marx PA, Burton DR (2009). Effective, low-titer antibody protection against low-dose repeated mucosal SHIV challenge in macaques. Nat Med.

[70] Gautam R, Nishimura Y, Pegu A, Nason MC, Klein F, Gazumyan A, Golijanin J, Buckler-White A, Sadjadpour R, Wang K (2016). A single injection of anti-HIV-1 antibodies protects against repeated SHIV challenges. Nature.

[71] Gautam R, Nishimura Y, Gaughan N, Gazumyan A, Schoofs T, Buckler-White A, Seaman MS, Swihart BJ, Follmann DA, Nussenzweig MC, Martin MA (2018). A single injection of crystallizable fragment domain-modified antibodies elicits durable protection from SHIV infection. Nat Med.

[72] Royce RA, Sena A, Cates W, Cohen MS (1997). Sexual transmission of HIV. N Engl J Med.

[73] Dunn DT, Newell ML, Ades AE, Peckham CS (1992). Risk of human immunodeficiency virus type 1 transmission through breastfeeding. Lancet.

[74] Cohen YZ, Lorenzi JCC, Seaman MS, Nogueira L, Schoofs T, Krassnig L, Butler A, Millard K, Fitzsimons T, Daniell X (2018). Neutralizing activity of broadly neutralizing anti-HIV-1 antibodies against clade B clinical isolates produced in peripheral blood mononuclear cells. J Virol.

[75] Caskey M, Klein F, Lorenzi JC, Seaman MS, West AP, Buckley N, Kremer G, Nogueira L, Braunschweig M, Scheid JF (2015). Viraemia suppressed in HIV-1-infected humans by broadly neutralizing antibody 3BNC117. Nature.

[76] Caskey M, Schoofs T, Gruell H, Settler A, Karagounis T, Kreider EF, Murrell B, Pfeifer N, Nogueira L, Oliveira TY (2017). Antibody 10-1074 suppresses viremia in HIV-1-infected individuals. Nat Med.

[77] Ledgerwood JE, Coates EE, Yamshchikov G, Saunders JG, Holman L, Enama ME, DeZure A, Lynch RM, Gordon I, Plummer S (2015). Safety, pharmacokinetics and neutralization of the broadly neutralizing HIV-1 human monoclonal antibody VRC01 in healthy adults. Clin Exp Immunol.

[78] Mayer KH, Seaton KE, Huang Y, Grunenberg N, Isaacs A, Allen M, Ledgerwood JE, Frank I, Sobieszczyk ME, Baden LR (2017). Safety, pharmacokinetics, and immunological activities of multiple intravenous or subcutaneous doses of an anti-HIV monoclonal antibody, VRC01, administered to HIV-uninfected adults: results of a phase 1 randomized trial. PLoS Med.

[79] Huang Y, Zhang L, Ledgerwood J, Grunenberg N, Bailer R, Isaacs A, Seaton K, Mayer KH, Capparelli E, Corey L, Gilbert PB (2017). Population pharmacokinetics analysis of VRC01, an HIV-1 broadly neutralizing monoclonal antibody, in healthy adults. MAbs.

[80] Gilbert PB, Juraska M, deCamp AC, Karuna S, Edupuganti S, Mgodi N, Donnell DJ, Bentley C, Sista N, Andrew P (2017). Basis and statistical design of the passive HIV-1 antibody mediated prevention (AMP) test-of-concept efficacy trials. Stat Commun Infect Dis..

[81] Mingozzi F, High KA (2011). Therapeutic in vivo gene transfer for genetic disease using AAV: progress and challenges. Nat Rev Genet.

[82] Balazs AB, Chen J, Hong CM, Rao DS, Yang L, Baltimore D (2012). Antibody-based protection against HIV infection by vectored immunoprophylaxis. Nature.

[83] Balazs AB, Ouyang Y, Hong CM, Chen J, Nguyen SM, Rao DS, An DS, Baltimore D (2014). Vectored immunoprophylaxis protects humanized mice from mucosal HIV transmission. Nat Med.

[84] Klein F, Halper-Stromberg A, Horwitz JA, Gruell H, Scheid JF, Bournazos S, Mouquet H, Spatz LA, Diskin R, Abadir A (2012). HIV therapy by a combination of broadly neutralizing antibodies in humanized mice. Nature.

[85] Horwitz JA, Halper-Stromberg A, Mouquet H, Gitlin AD, Tretiakova A, Eisenreich TR, Malbec M, Gravemann S, Billerbeck E, Dorner M (2013). HIV-1 suppression and durable control by combining single broadly neutralizing antibodies and antiretroviral drugs in humanized mice. Proc Natl Acad Sci USA.

[86] Klein F, Nogueira L, Nishimura Y, Phad G, West AP, Halper-Stromberg A, Horwitz JA, Gazumyan A, Liu C, Eisenreich TR (2014). Enhanced HIV-1 immunotherapy by commonly arising antibodies that target virus escape variants. J Exp Med.

[87] Diskin R, Klein F, Horwitz JA, Halper-Stromberg A, Sather DN, Marcovecchio PM, Lee T, West AP, Gao H, Seaman MS (2013). Restricting HIV-1 pathways for escape using rationally designed anti-HIV-1 antibodies. J Exp Med.

[88] Freund NT, Horwitz JA, Nogueira L, Sievers SA, Scharf L, Scheid JF, Gazumyan A, Liu C, Velinzon K, Goldenthal A (2015). A new glycan-dependent CD4-binding site neutralizing antibody exerts pressure on HIV-1 in vivo. PLoS Pathog.

[89] Freund NT, Wang H, Scharf L, Nogueira L, Horwitz JA, Bar-On Y, Golijanin J, Sievers SA, Sok D, Cai H (2017). Coexistence of potent HIV-1 broadly neutralizing antibodies and antibody-sensitive viruses in a viremic controller. Sci Transl Med.

[90] Julg B, Pegu A, Abbink P, Liu J, Brinkman A, Molloy K, Mojta S, Chandrashekar A, Callow K, Wang K (2017). Virological control by the CD4-binding site antibody N6 in simian-human immunodeficiency virus-infected rhesus monkeys. J Virol.

[91] Barouch DH, Whitney JB, Moldt B, Klein F, Oliveira TY, Liu J, Stephenson KE, Chang HW, Shekhar K, Gupta S (2013). Therapeutic efficacy of potent neutralizing HIV-1-specific monoclonal antibodies in SHIV-infected rhesus monkeys. Nature.

[92] Hessell AJ, Hangartner L, Hunter M, Havenith CE, Beurskens FJ, Bakker JM, Lanigan CM, Landucci G, Forthal DN, Parren PW (2007). Fc receptor but not complement binding is important in antibody protection against HIV. Nature.

[93] Lu CL, Murakowski DK, Bournazos S, Schoofs T, Sarkar D, Halper-Stromberg A, Horwitz JA, Nogueira L, Golijanin J, Gazumyan A (2016). Enhanced clearance of HIV-1-infected cells by broadly neutralizing antibodies against HIV-1 in vivo. Science.

[94] Lynch RM, Boritz E, Coates EE, DeZure A, Madden P, Costner P, Enama ME, Plummer S, Holman L, Hendel CS (2015). Virologic effects of broadly neutralizing antibody VRC01 administration during chronic HIV-1 infection. Sci Transl Med.

[95] Bar KJ, Sneller MC, Harrison LJ, Justement JS, Overton ET, Petrone ME, Salantes DB, Seamon CA, Scheinfeld B, Kwan RW (2016). Effect of HIV antibody VRC01 on viral rebound after treatment interruption. N Engl J Med.

[96] Scheid JF, Horwitz JA, Bar-On Y, Kreider EF, Lu CL, Lorenzi JC, Feldmann A, Braunschweig M, Nogueira L, Oliveira T (2016). HIV-1 antibody 3BNC117 suppresses viral rebound in humans during treatment interruption. Nature.

[97] Bar-On Y, Gruell H, Schoofs T, Pai JA, Nogueira L, Butler AL, Millard K, Lehmann C, Suárez I, Oliveira TY (2018). Safety and antiviral activity of combination HIV-1 broadly neutralizing antibodies in viremic individuals. Nat Med..

[98] Mendoza P, Gruell H, Nogueira L, Pai JA, Butler AL, Millard K, Lehmann C, Suárez I, Oliveira TY, Lorenzi JCC (2018). Combination therapy with anti-HIV-1 antibodies maintains viral suppression. Nature.

[99] Lynch RM, Wong P, Tran L, O’Dell S, Nason MC, Li Y, Wu X, Mascola JR (2015). HIV-1 fitness cost associated with escape from the VRC01 class of CD4 binding site neutralizing antibodies. J Virol.

[100] Sather DN, Carbonetti S, Kehayia J, Kraft Z, Mikell I, Scheid JF, Klein F, Stamatatos L (2012). Broadly neutralizing antibodies developed by an HIV-positive elite neutralizer exact a replication fitness cost on the contemporaneous virus. J Virol.

[101] Margolis DA, Gonzalez-Garcia J, Stellbrink H-J, Eron JJ, Yazdanpanah Y, Podzamczer D, Lutz T, Angel JB, Richmond GJ, Clotet B (2017). Long-acting intramuscular cabotegravir and rilpivirine in adults with HIV-1 infection (LATTE-2): 96-week results of a randomised, open-label, phase 2b, non-inferiority trial. Lancet.

[102] Bournazos S, Gazumyan A, Seaman MS, Nussenzweig MC, Ravetch JV (2016). Bispecific anti-HIV-1 antibodies with enhanced breadth and potency. Cell.

[103] Pace CS, Song R, Ochsenbauer C, Andrews CD, Franco D, Yu J, Oren DA, Seaman MS, Ho DD (2013). Bispecific antibodies directed to CD4 domain 2 and HIV envelope exhibit exceptional breadth and picomolar potency against HIV-1. Proc Natl Acad Sci USA.

[104] Lorenzi JC, Cohen YZ, Cohn LB, Kreider EF, Barton JP, Learn GH, Oliveira T, Lavine CL, Horwitz JA, Settler A (2016). Paired quantitative and qualitative assessment of the replication-competent HIV-1 reservoir and comparison with integrated proviral DNA. Proc Natl Acad Sci USA.

[105] Lengauer T, Sing T (2006). Bioinformatics-assisted anti-HIV therapy. Nat Rev Microbiol.

[106] Hake A, Pfeifer N (2017). Prediction of HIV-1 sensitivity to broadly neutralizing antibodies shows a trend towards resistance over time. PLoS Comput Biol.

[107] Rawi R, Mall R, Shen C-H, Doria-Rose NA, Farney SK, Shiakolas A, Zhou J, Chun T-W, Lynch RM, Mascola JR (2018). Accurate prediction of antibody resistance in clinical HIV-1 isolates. bioRxiv.

[108] Roopenian DC, Akilesh S (2007). FcRn: the neonatal Fc receptor comes of age. Nat Rev Immunol.

[109] Ko SY, Pegu A, Rudicell RS, Yang ZY, Joyce MG, Chen X, Wang K, Bao S, Kraemer TD, Rath T (2014). Enhanced neonatal Fc receptor function improves protection against primate SHIV infection. Nature.

[110] Gaudinski MR, Coates EE, Houser KV, Chen GL, Yamshchikov G, Saunders JG, Holman LA, Gordon I, Plummer S, Hendel CS (2018). Safety and pharmacokinetics of the Fc-modified HIV-1 human monoclonal antibody VRC01LS: a Phase 1 open-label clinical trial in healthy adults. PLoS Med.

[111] Ma JK, Drossard J, Lewis D, Altmann F, Boyle J, Christou P, Cole T, Dale P, van Dolleweerd CJ, Isitt V (2015). Regulatory approval and a first-in-human phase I clinical trial of a monoclonal antibody produced in transgenic tobacco plants. Plant Biotechnol J.

[112] Morris GC, Wiggins RC, Woodhall SC, Bland JM, Taylor CR, Jespers V, Vcelar BA, Lacey CJ (2014). MABGEL 1: first phase 1 trial of the anti-HIV-1 monoclonal antibodies 2F5, 4E10 and 2G12 as a vaginal microbicide. PLoS ONE.

[113] Veazey RS, Shattock RJ, Pope M, Kirijan JC, Jones J, Hu Q, Ketas T, Marx PA, Klasse PJ, Burton DR, Moore JP (2003). Prevention of virus transmission to macaque monkeys by a vaginally applied monoclonal antibody to HIV-1 gp120. Nat Med.

[114] Moog C, Dereuddre-Bosquet N, Teillaud JL, Biedma ME, Holl V, Van Ham G, Heyndrickx L, Van Dorsselaer A, Katinger D, Vcelar B (2014). Protective effect of vaginal application of neutralizing and nonneutralizing inhibitory antibodies against vaginal SHIV challenge in macaques. Mucosal Immunol.

[115] Veselinovic M, Neff CP, Mulder LR, Akkina R (2012). Topical gel formulation of broadly neutralizing anti-HIV-1 monoclonal antibody VRC01 confers protection against HIV-1 vaginal challenge in a humanized mouse model. Virology.

[116] Marrazzo JM, Ramjee G, Richardson BA, Gomez K, Mgodi N, Nair G, Palanee T, Nakabiito C, van der Straten A, Noguchi L (2015). Tenofovir-based preexposure prophylaxis for HIV infection among African women. N Engl J Med.

[117] Schoofs T, Klein F, Braunschweig M, Kreider EF, Feldmann A, Nogueira L, Oliveira T, Lorenzi JCC, Parrish EH, Learn GH (2016). HIV-1 therapy with monoclonal antibody 3BNC117 elicits host immune responses against HIV-1. Science.

[118] Haigwood NL, Montefiori DC, Sutton WF, McClure J, Watson AJ, Voss G, Hirsch VM, Richardson BA, Letvin NL, Hu SL, Johnson PR (2004). Passive immunotherapy in simian immunodeficiency virus-infected macaques accelerates the development of neutralizing antibodies. J Virol.

[119] Ng CT, Jaworski JP, Jayaraman P, Sutton WF, Delio P, Kuller L, Anderson D, Landucci G, Richardson BA, Burton DR (2010). Passive neutralizing antibody controls SHIV viremia and enhances B cell responses in infant macaques. Nat Med.

[120] Jaworski JP, Kobie J, Brower Z, Malherbe DC, Landucci G, Sutton WF, Guo B, Reed JS, Leon EJ, Engelmann F (2013). Neutralizing polyclonal IgG present during acute infection prevents rapid disease onset in simian-human immunodeficiency virus SHIVSF162P3-infected infant rhesus macaques. J Virol.

[121] Yamamoto H, Kawada M, Takeda A, Igarashi H, Matano T (2007). Post-infection immunodeficiency virus control by neutralizing antibodies. PLoS ONE.

[122] Yamamoto T, Iwamoto N, Yamamoto H, Tsukamoto T, Kuwano T, Takeda A, Kawada M, Tsunetsugu-Yokota Y, Matano T (2009). Polyfunctional CD4+ T-cell induction in neutralizing antibody-triggered control of simian immunodeficiency virus infection. J Virol.

[123] Nishimura Y, Gautam R, Chun TW, Sadjadpour R, Foulds KE, Shingai M, Klein F, Gazumyan A, Golijanin J, Donaldson M (2017). Early antibody therapy can induce long-lasting immunity to SHIV. Nature.

[124] Cohen YZ, Lorenzi JCC, Krassnig L, Barton JP, Burke L, Pai J, Lu CL, Mendoza P, Oliveira TY, Sleckman C (2018). Relationship between latent and rebound viruses in a clinical trial of anti-HIV-1 antibody 3BNC117. J Exp Med.

[125] Borducchi EN, Liu J, Nkolola JP, Cadena AM, Yu WH, Fischinger S, Broge T, Abbink P, Mercado NB, Chandrashekar A (2018). Antibody and TLR7 agonist delay viral rebound in SHIV-infected monkeys. Nature..

[126] Sogaard OS, Graversen ME, Leth S, Olesen R, Brinkmann CR, Nissen SK, Kjaer AS, Schleimann MH, Denton PW, Hey-Cunningham WJ (2015). The depsipeptide romidepsin reverses HIV-1 latency in vivo. PLoS Pathog.

[127] Azzoni L, Foulkes AS, Papasavvas E, Mexas AM, Lynn KM, Mounzer K, Tebas P, Jacobson JM, Frank I, Busch MP (2013). Pegylated Interferon alfa-2a monotherapy results in suppression of HIV type 1 replication and decreased cell-associated HIV DNA integration. J Infect Dis.

